# Certain Points Connected with Typhoid Fever

**Published:** 1876-02

**Authors:** F. C. Curtis


ART. II.—*Certain points connected with Typhoid Fever.* By Dr. F. C. CURTIS, M. D.*

It is not my intention to attempt to speak with any degree of fulness regarding typhoid fever, but rather simply to call attention to certain interesting phases and peculiarities of the disease which have fallen under my notice. The subject is one of interest at the present time, since the disease has prevailed to such a degree during the late summer and fall months that we may with propriety call it an epidemic.

The disease is, however, more or less endemic here, and sporadic cases occur constantly. I met with two or three cases in the spring months, and it must have been quite prevalent then, for I find by consulting the register of deaths since the last published report ending April 30th, which I have been permitted to do by the kindness of the city registrar, Mr. Dorlan, that eight deaths occurred from typhoid during the month of May. There was then a sudden falling off in the number of cases, three being reported respectively for June and July, and two in August. Then in September there were nine fatal cases, in October eight, and in November three, showing a rapid increase as the cool weather returned. It may be said that since April there has been an unusual tendency to disease of this type. In the last annual report of the city registrar, ending April 30, the whole number of deaths from typhoid is given as thirty-eight, about three times that number dying from scarlatina, and eleven from cerebro-spinal meningitis. In the year preceding there were but seventeen deaths from typhoid against one hundred and twelve from scarlatina and fifteen from spotted fever. The type of the disease this year has been rather mild according to my observation, and including the aborted and modified cases, which have been very numerous, the record of deaths does not present a fair exhibit of its degree of prevalence. I feel confident in saying that it has been unusually prevalent in our hands.

A search for the causes of this epidemic, if we may so call it, is attended with the usual obscurity of etiological investigations. It is seldom that one single cause operates to produce a disease; still, since typhoid fever is clearly not a prevalent disease in Albany,

*Read before the Medical Society of the County of Albany, Jan. 5th, 1876.

compared with other infectious diseases, naturally a special cause is looked for.

As is known to those who have read the very able discussion of the subject in Ziemssen's *Cyclopedia of Medicine,* by so distinguished an authority as Liebermeister, the possibility is there denied of the production of this disease otherwise than by a specific disease germ, it being maintained that while the decomposition of organic and excrementitious substances furnishes a soil particularly favorable for the development of these germs, it in no way originates the disease or becomes a focus of infection, except the germs be carried to it. This is at variance with the belief and former teachings of our most prominent observers and investigators into epidemics in this country. It precludes entirely the idea of its being propagated by contagion, as Watson teaches, and exposed swamp or pond bottoms, privies and clogged sewers, which have long been supposed, from the study of many epidemics in New England and elsewhere, to give rise to the disease by the simple reception into the system of their elements of decomposition, can only be considered to do so when the seed of this particular disease has germinated in them. The close observation and reasoning of German students has put to flight a great many time-honored doctrines, and I think this teaching will receive the assent of all when fully investigated.

It admits of a question, how much the use of our *river water* has to do with the increase of typhoid disease? The probability of this exerting a causative influence has certainly a degree of plausibility when we consider the manner in which it comes to us, and aside from the septic elements which the water no doubt contains, and also aside from the possibility of specific germs of disease entering the river throughout its length above this point, there is a peculiar facility for the stream to become the carrier of typhoid germs. It is well known that this disease is relatively much more prevalent on the other side of the river than this. Now, the main current of the river in its course down strikes the further shore at the upper part of Bath, thence it is deflected to this side, and coming very directly, reaches this side just at Quackenbush street. Directly at this point the city main, some three feet in diameter, projects six or eight feet in the full current, and the water thus received is forced by the pump into the Bleecker reservoir. It would appear, with the premises, that an unexceptional opportunity is here afforded for the reception of typhoid germs. I do not wish to record myself as condemning the use of this water, but simply to bring the matter before the society as a question which ought to receive our careful and unbiased attention. Its adoption was a questionable matter from the first, and gentlemen will remember well the almost unanimous feeling against it among scientific associations of this city. It was very properly brought officially, by vote of the Common Council, before this society for an opinion. The feeling was against it, but no vote was taken, as the subject was adjourned to another meeting, and meantime it was decided to adopt it. Now, after several months' use, it is a proper question to ask, the river being frozen over and navigation closed for a season, whether it has been a cause of ill health. If it be true that the germs of disease are carried directly to each house, ready for their destructive work, attempts at cleanliness and avoidance of foci for germination will avail very little to protect us. I believe the water was first thrown into the reservoir to any considerable extent about the first of August. In September and October we find typhoid fever, as shown by the death report, to be much increased in prevalence. I have seen it occurring in various parts of the city. It has, too, appeared not by any means among the poorer classes only, but in the houses of the well-to-do, which are well provided in all points regarding cleanliness and hygiene. I have known of cases appearing in some of the best drained houses of the city, situated on high ground that has been long built over. It has been impossible to trace any local cause in many cases, and in this statement I am sure I shall be borne out. This, and the fact of its very general distribution, suggest at least the possibility of a general cause, and I do not know of any other that would act so generally. It may be added, also, that other of the most fatal of our acute infectious diseases, scarlatina, diphtheria and cerebro-spinal meningitis, have been comparatively infrequent, judging by the registrar's report.

On the other hand, however, we find that typhoid fever prevailed in the spring, before the pumps were used. It is only fair, also, to allow that the mortality from all causes, or from zymotic diseases alone, has not been excessive since the water was introduced. Besides, typhoid has occurred outside of the region supplied by the Bleecker reservoir. We must allow that our experience has not been sufficient to condemn the use of this water; but, inasmuch as contaminated drinking-water is perhaps the most common source of typhoid fever, and it seems evident that the Hudson river is extremely favorable to contamination, I would withhold judgment till it can be more fully shown that Albany is not to become a second Munich.

*A point of interest* in the consideration of this disease is where shall we draw the line of distinction between it and cases of simple gastro-intestinal irritation. Every one has in mind the typical course of this fever, its gradual onset, the steady access of severity, passing in the second week to the brown-furred tongue, immobile expression and dusky hue in rather severe cases, with moderate diarrhœa, the petechial eruption, etc.; finally, after the third week, the gradual abeyance of symptoms and the long convalescence from the prostration induced by so general pathological change of all the tissues of the body. It has, no doubt, been the experience of every one here that during the fall we met with many cases that were evidently *essential* fevers, the fever of the case being independent of any organic lesion. There was sometimes diarrhœa, often the opposite condition, anorexia, furred tongue, headache and backache, lassitude and depression, and various other symptoms in the different cases, the feeling being on the part of the patient that he had a very "hard cold" or a "bilious attack," as the case might be. You probably apprehend the character of the affections I allude to. They are not *abortive typhoid*, which begins as an ordinary typhoid, but the duration of the disease is markedly shortened. If we accept the teachings of Liebermeister, we are to believe that many of these were cases of typhoid poisoning, the disease pursuing a mild form, either from the character or amount of the poison or the constitution of the recipient, just as in cholera epidemics a more or less trifling diarrhœa is very prevalent, or in the eruptive fevers we find all grades of severity. He even speaks of an "afebrile abdominal catarrh" due to typhoid infection, and has noted in some of them an enlargement of the spleen and the roseola faintly marked, constipation sometimes being present instead of diarrhœa. That many of these cases have actually been typhoid has sometimes been proved by the occurrence of intestinal hemorrhage, or some other alarming symptom, which may lead to an autopsy and the disclosure of typhoid lesions. A few cases are related of perforation of the intestine where symptoms were so insignificant as to be diagnosticated as simple intestinal derangement.

Liebermeister says that there is not a symptom of typhoid fever that is pathognomonic, and diagnosis is often difficult in cases that are not well marked, even from the general assemblage of symptoms. Dr. W. W. Johnson, in the last issue of the *American Journal of Medical Science*, proposes, "in order to reach a decision as to what is the feeblest manifestation of the typhoid infection: Does any combination of the most constantly associated symptoms, or does any one symptom, exist of necessity in typhoid fever? What is the most reliable symptom in the absence of all pathognomonic phenomena?" He weighs the various symptoms common to the disease severally and finds no one necessarily present in every case, even the most constant symptoms being frequently wanting. In regard to the symptom of fever, without which we can hardly conceive of the existence of the pathological processes which constitute the disease under consideration, and the absence of which particularly "so strips the disease of its familiar dress as to lose all semblance of its nature," he quotes from Liebermeister: "Typhoid fever in rare cases can arrive at advanced periods of its evolution and reveal itself suddenly by grave and even fatal accidents without the temperature, as determined by the thermometer, sensibly departing from the limits of the normal state." He then gives the notes of eight cases of varying degree of severity, but characterized for the most part by lassitude, slight headache, coated tongue, constipation, and followed by considerable emaciation, having a course of several weeks and the characteristic relation of morning and evening temperature, but of low degree, being in one case no higher than 99° in the morning.

Such cases, it seems to me, hardly admit of doubt as to their nature, and I think we have always been accustomed, in this neighborhood, to regard them as typhoid. Dr. Gile, of Columbia county, an old, experienced practitioner, told me that he had always looked upon cases of continued fever lasting for ten days as typhoid. The question which I wish to propose is whether the numerous cases of a much milder disease which we met with last fall were of the same nature—hardly necessitating confinement to bed, having a gradual onset; there was dull headache and back-ache, furred tongue, anorexia, diarrhœa or constipation, more usually the latter, frequently a rather dusky hue to the face even when the patient kept about, the case not yielding readily to ordinary measures addressed to stomachic correction, but holding on for ten days or a fortnight, or even longer sometimes, before a return to health. I have met with three cases in one house and family. (By way of parenthesis, I will say here that in one of these cases there was abscess of the tongue, the only case of the kind I have ever met with, and showing a depraved condition of the system.)

Now, to claim the slightest connection between such cases and typhoid fever is to go counter to the explicit teachings of some most respected authorities. Trousseau, discussing the value of thermometric observations in this disease, quotes approvingly from Wunderlich: "When on the first or second day the morning temperature is 40° Centigrade or 104° Fahr., the disease is not typhoid; and when by the evening of the fourth day the temperature has not attained 103.1° Fahr., the disease is not typhoid." I am positive I speak the opinion of all who hear me in saying that the disease is not to be confined in such narrow limits, and rather that where the temperature reaches so high a range we have to deal not with a mild case, as Liebermeister says, but one of grave importance. Our form of the disease must differ from that occurring in Europe. I see reason to believe that cases such as I have spoken of, occurring as they do during the prevalence of typhoid fever, the occurrence of two or more cases in the same locality, the feeble manifestation of one or more symptoms of typhoid, or the exhibition of a general physiognomy similar to that of our endemic continued fever in a dwarfed form considering, too, that we *know* that the poison of typhoid has a very variable degree of virulence in different localities or individuals, may we not believe that many of these cases are due to the poisonous influence of typhoid bachteria, and not to be called simple continued fever, febricula, bilious or gastric fever, or malarial, or simply gastro-intestinal irritation.

Where we shall draw the line even lowered so far, however, it will be difficult to say; for these disturbances in the functions of the system shade into each other until we come to those which may with all propriety be called ephemeral.

In this connection I would say a word concerning certain unusual cases, evidently of the nature of Typhoid fever, but showing a departure from the usual course of symptoms. One of these I will give: Ellen F., a chambermaid, æt. 20, living in a healthy locality in the immediate neighborhood of which no Typhoid fever had occurred—an anæmic girl, subject to occasional periods of malaise, lasting two or three days—was taken with what she regarded as one of her usual attacks of general malaise on the 24th of September. This continued for four days when she began to experience pain, intermittent in character, on the left side about the margin of the ribs, not so severe as to prevent sleep the night following, but next day became more severe and extended across the abdomen, being still intermittent. The bowels moved naturally, and there was no vomiting though nausea was present. Through the night following, the 29th, pain was very severe she said, allowance being made for her nervous temperament. She was seen early next morning, her countenance was rather anxious, face pale, decubitus dorsal with legs extended, pain described as most severe below the ribs on left side and extending across the abdomen.

Tenderness of abdomen was not marked. Half gr. doses of opium every hour relieved her by afternoon, and during twelve hours of the night only two grs. were taken, and there was little pain until next day at 2 o'clock P. M. During this quiet period her pulse was about 94. As the pain returned the pulse ran up and there was a good deal of heat of skin. This subsided early in the night, aconite and sweet spirits of nitre being given, and she slept pretty well. Next day, October 2d, pain again found reduced, the tongue was rather dry and brownish but not furred—no headache—pulse 100 and full; temperature 100° F.; Resp. 24; evening temperature 101° F. Abdomen soft but somewhat distended and tympanitic. Bowels hard, not moved for three days, probably from the effects of opium of which only a small quantity was now called for, and a cathartic was ordered, which acted freely next day. Next morning, the 10th since the onset, pulse was 110; temperature 100.7° F.; R. 25. She had an apathetic look and there was a constant flush on one cheek; mind perfectly clear; tongue of natural size, slightly coated with brown fur; breath had a disagreeable odor; pain still present on moving or inspiring deeply; evening temperature was 102° F. I had little doubt that the case was one of Typhoid. She was stimulated freely and sent to St. Peter's Hospital, when she came into the service of Dr. Hun. Her pain was so acute on the left side that he believed at first that it was a case of pleurisy, and for a time she was greatly reduced. She remained in for six or eight weeks with low adynamic symptoms, and having, I believe, a good deal of the pain she early showed, with temperature as Dr. Kilbourne, the house physician, tells me, showing an evening exucerbation of 102°, with morning remission.

I saw her after leaving the hospital; she was then much emaciated, and was still pale and weak, and has not yet fully recovered her strength. Judging from the thermometric range, the general course of the disease, its adynamic character and the emaciation following, I believe that this was an essential Typhoid fever, although the local pain, the cause of which I cannot account for, seemed much of the time so marked as to lead to the belief that it was entirely dependent upon a serous inflammation.

I saw another patient, a boy, ten years of age, under the care of Dr. Morgan, whose case appeared to be one of Typhoid, departing from its ordinary type. Here attention was at first entirely directed to the brain, the symptoms all pointing to meningeal inflammation, But as these gradually passed away leaving only a degree of stupor, the low adynamic condition of Typhoid fever developed, running a complete, though mild course, ending in the usual long stage of convalescence so constant with Typhoid. Irregular cases such as these make diagnosis difficult. Still although authors may agree that there is not a single symptoms of Typhoid fever that is pathognomonic, yet there is something in the character of the disease, its physiognomy to use an expressive word of Dr. Wm. H. Draper's,—the Typhoid symptoms, the course the disease takes, etc., which even in masked and complicated cases speak as to the nature of the affection. All diseases are not by any means typhical in their manifestations, and we are not to look for an unvarying sequence of symptoms to follow the reception of a specific poison into the system. An author speaks of the specific typhoid poison as producing *diseases* (using the plural) which differ among themselves, some being so serious that life is almost inevitably destoyed by them, others so trifling that patient and physician are in doubt whether there really is any disease at all. To be sure, we meet with many cases which are purely typical, but we are not justified, in the light of pathological investigation, in recognizing no diseases which depart from this type as related to them, and we are again and again forced to the belief, even from the clinical development alone of cases, that there are manifold forms in which Typhoid fever develops itself in different cases.

One case which I met with during the fall occurred in the person of a man 75 years of age. I speak of this as an unusual case, the disease as a rule affecting the middle aged. The subject was a remarkably rugged man, and this his fatal sickness was his first serious one. He died in the sixth week of asthenia, the disease being attended with marked depression almost from the first, the tongue being dry as parchment, and the mind wandering, and a stupor soon coming on from which he could hardly be aroused. It occurred to me in this case as it has before in others that when the vital powers are blunted by age there is not the acute sensibility to morbific impressions that there is at middle life when they are keenest, and if these impressions are made somewhat gradually they are frequently resisted, or rather fail to affect, and the patient lives on regardless of them as it were. I have noted the same thing in very young infants sometimes, they exhibiting a tenacity to life that was very remarkable, and recovering when it seemed as if life had almost entirely gone out of them.

I have met with two or three cases which showed the deleterious effects of Typhoid fever upon the circulatory apparatus. One of these was a case of fatal weakening of the heart. The patient was a middle aged German, of small stature and spare frame, by occupation a cabinetmaker. In the latter part of August he was taken with mild initiatory symptoms of Typhoid fever, which developed gradually into a rather mild type of the disease. A dry, glazed condition of the tongue, some muscular tremulousness and slight wandering of the mind constituted the worst manifestations of the disease. By the end of September convalesence began, and a week later he was sitting up part of the day, and was in a fair way for recovery, until the middle of October, when some indiscretion on his part, probably eating a quantity of grapes without removing the pits, brought on a relapse, and a return of the old symptoms. There was no marked weakness of the pulse, nor had there been previously. On the morning of the third day of the relapse he was found to have had a very restless night, and there was a decided weakening of the heart, the pulse being rapid, quick and small. Stimulants in large quantity were at once ordered, but no effect was produced by them. The pulse became more and more thready through the day, the first heart sound was faint and he became weaker, the voice sinking to a whisper. Aside from weakness there were no marked additional symptoms. The mind was quite clear, there was no dyspnoea, and the patient had no thought of his approaching dissolution. He died about twenty-four hours after it was discovered that this weakening of the heart had set in. It is a point of interest that this condition should arise in a case which may fairly be considered a mild one, and aside from any exciting cause. It leads me to discredit the teaching of some that degeneration of the muscular tissue of the heart is produced in this disease as a direct result of the *fever heat,* and long continued elevation of temperature.

Another case of a somewhat similar nature, one of venous thrombosis, I saw a little later. Being irregular in its onset it was not recognized at first as a case of Typhoid fever. The patient was a young man, of spare habit of body, by occupation a clerk. His sickness was traceable to a clogged sewer running under the store in which he was employed on Broadway. He was taken with very acute cephalalgia, continuing as the almost only symptom for a week or more. Symptoms more decisive of Typhoid fever developed gradually. Quite early in its course a rapid, weak, dicrotic pulse showed itself, and also with it the prostration peculiar to the disease. His treatment was tonic and stimulating throughout. He was confined entirely to his bed for about ten weeks. In the sixth week he was taken with chill, and severe pain in the calf of the left leg, followed directly by swelling of the foot, ankle and leg. Hot fomentations were applied with the effect of releiving the pain in good measure, but the swelling continued for two months, and there was after a little pain about the knee on attempting to straighten the limb which was held in a flexed position. There was no marked tenderness on pressure behind the knee joint. It is evident that in this case thrombus form in the popliteal vein, not higher than this for no enlargment of the saphenous could be detected, and there was no swelling above the knee the condition is a rare one. Liebermeister reports thirty-one cases of thrombi in 1743 cases of Typhoid, but one of which affected the popliteal vein. He says farther of it that it usually occurs during the period of convalescence, that the majority of cases are amongst men, and that it is much more frequent on the left side than the right, conditions which correspond with those obtaining in my case. The circumstance of its more frequent occurrence upon the left side is explained by the fact that the left common iliac vein being crossed by the right common iliac artery does not admit of so free a flow of the current of blood in this vessel as in that of the opposite side. The prognosis is good, death having been caused by it only by the occurrence of detachment of a portion of the clot, causing embolism of the pulmonary artery. In my case the recovery is now complete excepting slight swelling about the ankle which still remains.*

*Feb. 7th. He is still troubled with a swelling of the leg and foot, with pain in the part, since getting about his business again.

The same patient showed still another symptom of affection of the circulatory apparatus. Not long after the occurrence of thrombosis, while still in the extremely prostrated condition of his early convalescence, he was taken with chill following a little attempt at exercise, and with it rapid breathing and dyspnoea. The pulse became very frequent, quick and weak, and with its dicrotism could hardly be counted. I might remark here that he had throughout his disease a troublesome cough coming on with the weakening of the pulse. It was believed that his dyspnoea was due to impending paralysis of the heart, not to pulmonary embolism. Heat was applied to the extremities, and stimulants ordered very freely. A wineglassful of whiskey, nearly 2 ozs. was given every two hours, and a subsequent attack coming on a few days later it was given every hour, with the effect of relieving the dyspnoea and bringing down the rapid pulse. There were three or four attacks of this character.

In regard to the use of alcoholics in Typhoid fever, I have seen reason to agree fully with Dr. W. H. Thomson, of New York, who in three very interesting lectures reported in the RECORD, enforces the teaching of Graves on this point, that is to give them only as the condition of the heart calls for them. In regard to the manner of giving, he says: "The dose must be such always as to increase the cardiac systole, and a few free doses are better than many small ones." I am fully persuaded that they should not be given in the early stage of the disease, before its weakening effect upon the system calls for a spur. I believe that it is only a spur, and not to be used until indicated by flagging of the vital powers. Still the pulse is not the only evidence of this, for no one would fail to begin with whiskey when the tongue becomes dry, sordes collect and the face becomes immobile with the Typhoid expression or lack of expression.

The value of thus abstaining I saw illustrated in a case of the disease at St. Peter's Hospital in September. The case was one of the most perfectly classical that we usually find, in its onset, course and manifestation of most of the peculiar symptoms. The lenticular eruption in particular was very abundant.

The patient, a woman, æt. 40, a domestic living on Elk street, was brought in during the second week. It was a case of moderate severity. A grain of quinine every four hours comprised the treatment; no stimulants were given. During the third week, the tongue rather abruptly became dry and brown, muttering delirium came on and the general symptoms of the low condition of Typhoid poisoning ensued. A few decided doses of whiskey were at once given and very soon showed their effects, as she speedily rallied from this state, began to convalesce and made a very quick recovery. I do not believe that if she had been given whiskey at once from the time of entering the hospital that she would have felt the spurring that was called for, and there is no probability that she would have progressed in so happy a manner through the convalescing period of her disease.

The whole subject of Typhoid fever has appeared to me to attract an unusual degree of attention of late. A year ago the article by Liebermeister appeared in Ziemssen's Cyclopedia, full of new ideas. No one can study it without being impressed with the evidence of work and close observation. It has seemed that since then hardly an issue of a medical journal has appeared without more or less upon the subject.

So much study and investigation cannot fail to produce knowledge, and the more light is thrown upon this or any other subject, the more will erroneous ideas long held, give way to true ones and new facts be developed and given to us.

